# Transcriptomic analysis reveals partial epithelial–mesenchymal transition and inflammation as common pathogenic mechanisms in hypertensive nephrosclerosis and Type 2 diabetic nephropathy

**DOI:** 10.14814/phy2.15825

**Published:** 2023-10-09

**Authors:** Ole Petter Nordbø, Lea Landolt, Øystein Eikrem, Andreas Scherer, Sabine Leh, Jessica Furriol, Terje Apeland, Piotr Mydel, Hans‐Peter Marti

**Affiliations:** ^1^ Department of Clinical Medicine University of Bergen Bergen Norway; ^2^ Department of Medicine, Haugesund Hospital Helse Fonna Haugesund Norway; ^3^ Department of Medicine Haukeland University Hospital Bergen Norway; ^4^ Department of Clinical Science University of Bergen Bergen Norway; ^5^ Spheromics Kontiolahti Finland; ^6^ Department of Pathology Haukeland University Hospital Bergen Norway; ^7^ Stavanger University Hospital Stavanger Norway

## Abstract

Hypertensive nephrosclerosis (HN) and Type 2 diabetic nephropathy (T2DN) are the leading causes of chronic kidney disease (CKD). To explore shared pathogenetic mechanisms, we analyzed transcriptomes of kidney biopsies from patients with HN or T2DN. Total RNA was extracted from 10 μm whole kidney sections from patients with HN, T2DN, and normal controls (Ctrl) (*n* = 6 for each group) and processed for RNA sequencing. Differentially expressed (log_2_ fold change >1, adjusted *p* < 0.05) genes (DEG) and molecular pathways were analyzed, and selected results were validated by immunohistochemistry (IHC). ELISA on serum samples was performed on a related cohort consisting of patients with biopsy‐proven HN (*n* = 13) and DN (*n* = 9), and a normal control group (*n* = 14). Cluster analysis on RNA sequencing data separated diseased and normal tissues. RNA sequencing revealed that 88% (341 out of 384) of DEG in HN were also altered in T2DN, while gene set enrichment analysis (GSEA) showed that over 90% of affected molecular pathways, including those related to inflammation, immune response, and cell‐cycle regulation, were similarly impacted in both HN and T2DN samples. The increased expression of genes tied to interleukin signaling and lymphocyte activation was more pronounced in HN, while genes associated with extracellular matrix organization were more evident in T2DN. Both HN and T2DN tissues exhibited significant upregulation of genes connected with inflammatory responses, T‐cell activity, and partial epithelial to mesenchymal transition (p‐EMT). Immunohistochemistry (IHC) further confirmed T‐cell (CD4^+^ and CD8^+^) infiltration in the diseased tissues. Additionally, IHC revealed heightened AXL protein expression, a key regulator of inflammation and p‐EMT, in both HN and T2DN, while serum analysis indicated elevated soluble AXL levels in patients with both conditions. These findings underline the shared molecular mechanisms between HN and T2DN, hinting at the potential for common therapeutic strategies targeting both diseases.

## Graphical abstract

Although no single gene was dysregulated between hypertensive nephropathy (HN) and Type 2 diabetic nephropathy (T2DN), some differences were observed. Genes distinctly expressed in either HN or Type 2 diabetic nephropathy, a network related to toll‐like receptor signaling emerges in HN, while processes relating to ECM organization are more prevalent in T2DN. Further, the M1 macrophage phenotype was only observed in T2DN.

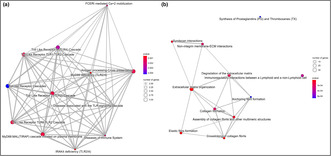

## 1 INTRODUCTION

Chronic kidney disease (CKD) affects about 10% of the global adult population. The leading causes of CDK are DM and HT, with rising prevalence rates worldwide (Honeycutt et al., 2013; Jha et al., 2013). Omics and systems biology technologies are widely used to identify novel biomarkers and therapeutic targets in renal disease (Berthier et al., 2017; Cisek et al., 2016; Rhee, 2018), for example, transcriptomes from biopsies of patients with autoimmune diseases affecting the kidneys and diabetic nephropathy have been widely reported (Arazi et al., 2019; Der et al., 2019; Rudnicki et al., 2015; Woroniecka et al., 2011). Results from these studies have helped to unravel the complexity of renal immune infiltrate associated with lupus nephropathy (Arazi et al., 2019) and emphasize the role of inflammation in diabetic kidney disease (DKD), irrespective of the type of diabetes and the patient's ethnic background (Rudnicki et al., 2015; Woroniecka et al., 2011). Moreover, transcriptomic analysis of kidney allografts has allowed the identification of gene signatures associated with rejection or tolerance, and it has helped to clarify the previously underestimated role of the innate immune system in transplant rejection (Gallon et al., 2018; Halloran et al., 2018; Mueller et al., 2019).

However, although hypertensive nephrosclerosis (HN) represents the second leading cause of CKD, renal transcriptomes from patients with HN have not been analyzed in similar detail (Chen et al., 2016). Moreover, unfortunately, a comparative evaluation of HN and Type 2 diabetic nephropathy (T2DN) transcriptomes has not been performed so far. However, this analysis could help to reveal shared disease progression mechanisms and, most importantly, suggest common treatments for the two leading causes of CKD.

To fill this knowledge gap and to obtain new insights into underlying pathogenetic pathways. In the present study, we comparatively analyzed RNA sequencing and immunohistochemistry (IHC) renal biopsies from patients diagnosed with HN or T2DN and from a control group without significant pathological features. We report highly similar gene expression profiles in HN and T2DN. Most importantly, overexpression of genetic pathways associated with inflammation, partial epithelial–mesenchymal transition (p‐EMT), extracellular matrix (ECM), and tissue remodeling are detectable in both diseased tissues.

## 2 METHODS

### 2.1 Patients and kidney biopsies

Patients were identified through the Norwegian Kidney Biopsy Registry (NKBR). We selected FFPE renal biopsies from patients with

1. HN without other comorbidities (*n* = 6),
2. T2DN (*n* = 6), and
3. normal or nonsignificantly altered renal tissue (*n* = 6) from patients who had undergone a biopsy due to suspected kidney disease. However, pathologists found no evidence of pathological changes in their tissue.

Inclusion criteria for diabetic and hypertensive patients were as follows: adult patients (>18 years) from our local Western Norwegian Health Region (Helse Vest) with an estimated glomerular filtration rate (eGFR) ≥ 30 mL/min/m^2^ and no other systemic diseases (e.g., lupus erythematosus, vasculitis), or other primary renal disorders. Kidney biopsies containing sparse, nonrepresentative amounts of tissue or only minimal histological alterations were excluded.

For the control biopsies, the inclusion criteria were normal serum creatinine levels with normal eGFR in the absence of arterial hypertension, diabetes, or systemic disease. Electronic patient charts were meticulously reviewed to exclude control patients who later developed kidney disease, ensuring the integrity of our control cohort.

The same inclusion criteria were applied for patient selection for ELISA analysis. The Regional Ethics Committee of Western Norway approved these investigations (REK vest no. 2013/553 for tissue analysis and REK vest no. 609670 for serum analysis). All participants provided written informed consent before inclusion.

### 2.2 RNA extraction

Total RNA was extracted from whole FFPE renal biopsy sections (10 μm thickness) using the High Pure FFPE‐tissue RNA Isolation kit (catalogue no. 06650775001; Roche Holding AG), as previously described (Eikrem et al., 2016; Landolt et al., 2016). Notably, formalin fixation of tissue, while preserving tissue integrity and protein structures, might alter RNA, as nucleobases are chemically modified by adding mono‐methylol groups and adenine dimerization (Mason & O'Leary, 1991; Rait et al., 2004). Biopsy storage and conditions, fixation time, and specimen size also affect RNA quality (Ahlfen et al., 2007). The primary metric used to assess the quality of RNA derived from FFPE tissues is the DV200 value (percentage of RNA with >200 nucleotides). A DV200 value above 70% is considered good, while values between 30% and 70% require increasingly higher RNA quantity for library preparation in RNA seq. The quality and quantity of extracted RNA were assessed using Agilent RNA 6000 Nano Kit on a 2100 Bioanalyzer instrument (Agilent Technologies).

### 2.3 RNA sequencing and read assembly

RNA inputs of 38–1324 ng total RNA per sample were used for library preparation using the TruSeq RNA Access Library Preparation Kit (Illumina, Inc.). RNA sequencing was performed on an Illumina NextSeq500 system (Illumina, Inc.) as 75‐bp paired ends at the Genomics Core Facility, Norwegian University of Science and Technology (NTNU). Assembled reads were aligned to the Homo sapiens hg38 reference genome using Gencode (https://www.gencodegenes.org/) (Frankish et al., 2019). The gene expression data were deposited into the Gene Expression Omnibus database (GSE166239).

### 2.4 Immunohistochemistry

FFPE sections (3 μm) were deparaffinized in xylene and rehydrated using decreasing ethanol concentrations. For AXL and VIM staining, sections were rinsed in distilled water and heated in pH 6 target retrieval buffer (catalogue no. S1699; Dako) in a microwave oven for 30 min. For CD4 and CD8 staining, sections were similarly rehydrated and microwave heated, but a pH 9 buffer (catalogue no. S236784‐2; Dako, Glostrup, Denmark) was used for antigen retrieval. The following primary antibodies were targeting CDH1 (dilution 1:3000, catalogue no. AF748, R&D Systems); AXL (dilution 1:1000, catalogue no. AF854, R&D Systems); VIM (dilution 1:1000, catalogue no. ab92547); CD4 (dilution 1:20, clone 4B12, catalogue no. M7310); CD8 (dilution 1:100, clone C8/144B catalogue no. M7103, Dako). A Purified Rabbit Anti‐Goat antibody (catalogue no. 6164‐01; Southern Biotech) was applied for 30 min. Bound antibodies were detected by incubating sections with envision rabbit–horseradish peroxidase (catalogue no. K400011‐2; Dako) for 30 min at room temperature. Sections were counterstained with hematoxylin, dehydrated in ascending alcohol concentrations and xylene, and cover‐slipped using a nonaqueous mounting medium. The stained samples were examined using Aperio eSlide Manager (Leica Biosystems) and protein abundance estimated from strong positive pixel counts to using the Aperio ImageScope 9.1 software (Leica Biosystems). Strong positive pixel count was used to minimize the effect of artifacts.

### 2.5 ELISA assay

Serum AXL was quantified using Human Axl DuoSet® ELISA (Cat no. DY154, R&D Systems) using commercially available ancillary products (Cat no. DY008: DuoSet ELISA Ancillary Reagent Kit 2), according to the manufacturer's protocol.

### 2.6 Transcriptomic data visualization

Data visualization was carried out using ggplot2 (Wickham, 2016) and FactoMineR for principal component visualization (Lê et al., 2008). Hierarchical clustering was performed using the complete linkage method, and heatmaps were constructed using the complexHeatmap package (Gu et al., 2016).

### 2.7 Transcriptomic EMT score

EMT score was determined for each transcriptome using a previously described method (Tan et al., 2014) and recently made available as an R‐package (Chakraborty et al., 2020). The method was originally developed to assess EMT enrichment in bladder, breast, colorectal, gastric, lung, and ovarian cancers. The input data (EMT marker genes) consisted of generic EMT genes from tumor and cell lines (Tan et al., 2014). A two‐sample Kolmogorov–Smirnov test was performed to assess EMT enrichment extent. Samples with a low enrichment score (−1 minimum) displayed a predominantly epithelial‐like gene expression pattern. In contrast, a higher EMT score (+1 maximum) was consistent with a shift toward a mesenchymal transcriptomic profile.

### 2.8 Scoring of morphological changes

The pathologist conducted evaluations of several anatomical changes including interstitial fibrosis, arteriolosclerosis, arteriosclerosis, and tubular atrophy. In assessing interstitial fibrosis, a numerical scoring system of 0–3 was employed. Here, a score of 0 indicated less than 5% of the cortical area was affected. A score of 1 signified an affected area between 5% and 25%. A score of 2 represented a 25%–50% affected area, and a score of 3 indicated that more than 50% of the area was affected. Other morphological alterations were also scored, but on a scale from 1 to 4, with 1 meaning “not present,” 2 denoting “slight,” 3 for “moderate,” and 4 signifying “severe” changes.

### 2.9 Statistical analysis

Statistical analysis of differentially expressed genes (DEG) was performed using DESeq2 (Love et al., 2014), with the patient's age as a confounder when constructing the design matrix. Only transcripts with counts >10 were included in the analysis. Ingenuity pathway analysis (IPA) to detect enriched biological pathways in the gene sets was implemented using a knowledge base of manually curated gene sets (Qiagen, Inc., Hilden, Germany). Fisher's exact *t*‐test was used to evaluate gene overlap in test and curated datasets for curated gene or gene sets. The FGSEA analysis (Sergushichev, n.d.) was performed with 1000 permutations with the MSigDB version 7.2 Hallmarks gene set and the “msigdb.v7.2.symbols”‐ GMT file (containing all the gene sets annotated in MSigDB). Ranks for each of the transcripts were computed as ‐log_10_, where is the log_2_ fold change in gene expression for each gene and is the false discovery rate. DEGs correlated to morphological tissue changes were determined using a Spearman correlation test. To evaluate protein abundance from IHC data, we employed a Kruskal–Wallis test to determine the overall differences among groups, and pairwise Wilcoxon tests for direct comparisons between specific groups. The data used for these tests were log_10_‐transformed values of dividing the strong positive pixel count (Nsp) by the total pixel count (Ntotal), thus providing a measure of protein abundance relative to the total area analyzed.

## 3 RESULTS

### 3.1 Study description

Patient characteristics are summarized in Table 1. Control subjects had normal blood pressure and eGFR, and they were, on average younger than patients with T2DN or HN (average age: 35, vs. 59 and 56 years, respectively, *p* < 0.01). Metrics for RNA purification, including the total amount of RNA, DV200 values, and RNA integrity numbers (RIN), are provided in Table S1. Additional clinicopathological data are provided in Table S2.

**TABLE 1:** Patient characteristics.

Patient ID	Age (years)	Gender	Hypertension	Systolic BP	Diastolic BP	Diabetes	eGFR (mL/min/m^2^)	Proteinuria (g/day)	ARB/ACEI
Ctrl 1	34	Male	No	130	90	No	89	0.00	Non
Ctrl 2	37	Female	No	116	65	No	104	1.00	Non
Ctrl 3	22	Female	No	114	65	No	71	0.03	Non
Ctrl 4	39	Male	Yes	138	88	No	113	0.29	ARB
Ctrl 5	48	Male	Yes	150	93	No	92	0.00	Non
Ctrl 6	33	Male	No	120	80	No	96	0.05	Non
T2DN 1	53	Male	Yes	150	85	Yes	44	4.70	ARB
T2DN 2	42	Male	Yes	120	85	Yes	81	2.43	ACEI
T2DN 3	81	Female	Yes	210	80	Yes	47	10.60	ACEI, ARB
T2DN 4	57	Male	Yes	160	85	Yes	49	21.00	Non
T2DN 5	59	Male	Yes	140	80	Yes	33	0.00	Non
T2DN 6	65	Female	Yes	190	70	Yes	33	1.75	ARB
HN 1	59	Male	Yes	157	115	No	56	0.20	Non
HN 2	58	Male	Yes	240	120	No	54	0.24	ARB
HN 3	57	Male	Yes	150	100	No	47	1.10	Non
HN 4	60	Male	Yes	147	91	No	50	0.00	Non
HN 5	50	Male	Yes	142	90	No	90	2.00	Non
HN 6	55	Male	Yes	165	80	No	65	0.50	Non

*Note*: Patient features at time of biopsy. Total number of biopsies = 18, T2DN = type 2 diabetic nephropathy. Ctrl = controls. HN = hypertensive nephrosclerosis. BP = blood pressure, eGFR = estimated glomerular filtration rate. eGFR was calculated with the CKD‐EPI formula. Proteinuria was measured as grams per day (g/d), ARB = angiotensin receptor blocker, ACEI = angiotensin‐converting‐enzyme inhibitor. Blood pressure measurements at day of biopsy. Hypertension column indicates if the patient had known hypertension.

### 3.2 Differentially expressed genes

**FIGURE 1:**
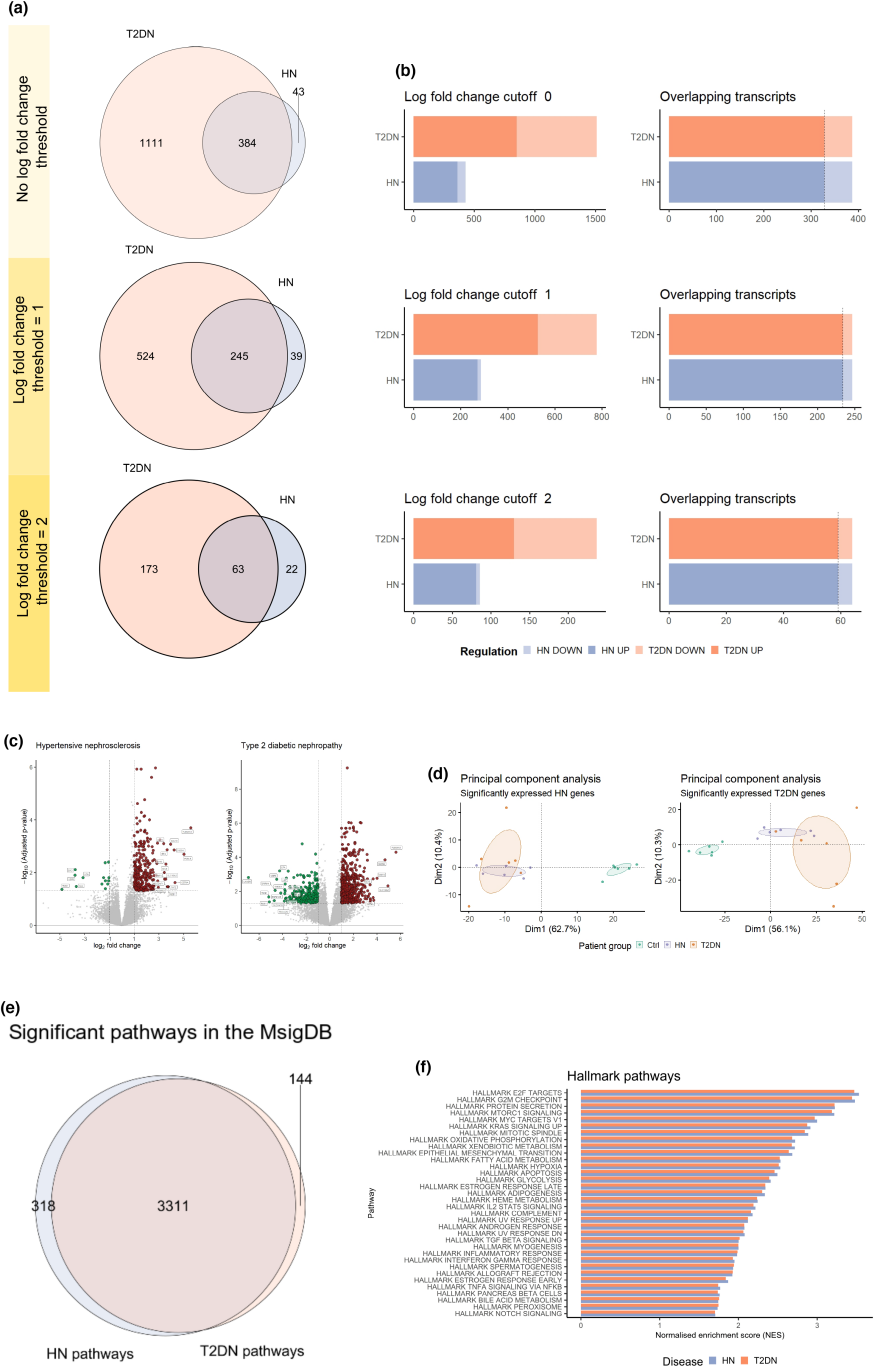
Hypertensive nephropathy and diabetic nephropathy share similar transcriptomic profiles. (a) Venn diagram depicting overlap and unique expression of genes for T2DN nephropathy (orange) and hypertensive nephropathy (violet). (b) The total number of differentially expressed genes in T2DN and HN is shown as upregulated (solid colors) and downregulated (transparent) in both disease entities. (c) Volcano plot showing the distribution of gene expression in HN (left) and T2DN (right). The 20 most dysregulated genes are highlighted. (d) Principal component analysis based on differentially expressed HN genes (Dim1: 62.7% and Dim2: 10.4% separation) and T2DN genes (Dim1: 56.1% and Dim2: 10.3% separation). (e) Overlapping and distinct outputs for gene set enrichment analysis for HN and T2DN show a total of 6637 shared gene sets between HN and T2DN, 162 distinct for HN and 257 for T2DN. (f) Gene set enrichment analysis based on the Hallmark dataset from the Molecular Signatures Database shows that the annotated gene sets have similar normalized enrichment scores. PCA inputs: All transcripts with an adjusted *p*‐value < 0.05. Patient groups coloring: HN; violet, T2DN; orange, Ctrl; turquoise. Patients: *n* = 6 per group.

In our analysis, HN tissues exhibited a low number of DEG compared to T2DN tissues (Figure 1a). This might reflect a heightened disease burden in T2DN, potentially due to the combined effects of hypertension and diabetes mellitus. Notably, regardless of the fold change thresholds employed, 88% (341 out of 384) of the DEG identified in HN were also differentially expressed in T2DN specimens. No significant gene expression alterations were observed between hypertensive nephrosclerosis and diabetic nephropathy (Figure 1b). A noteworthy observation was the limited number of transcripts that were downregulated in HN. In contrast, T2DN specimens exhibited a balanced distribution of up‐ and downregulated DEGs (Figure 1c). Furthermore, while there are evident similarities in the gene expression profiles of HN and T2DN, the principal components analysis (PCA) distinctly separated the diseased tissues from the controls along the primary axis, principal component 1 (PC1) (Figure 1d). Age was factored as a covariate in the analysis due to the discernible differences between the control and disease groups.

To elucidate potential differences in gene expression profiles, we performed gene set enrichment analysis (GSEA) using the FGSEA algorithm. We found that >90% of involved molecular pathways were similarly affected in HN and T2DN tissues (Figure 1e,f). Several similarly highly enriched pathways, reported in Figure 1f, including E2F Targets, G2M Checkpoint, MYC Targets, and Mitotic Spindle, were related to cell‐cycle regulation. However, the expression of genes included in a variety of pathways associated with inflammation and immune response, such as “IL2 STAT5 signaling,” “Complement,” “Inflammatory response,” “Interferon‐gamma response,” and “TNFA signaling,” was also increased in both HN and T2DN samples.

### 3.3 Pathway analysis

**FIGURE 2:**
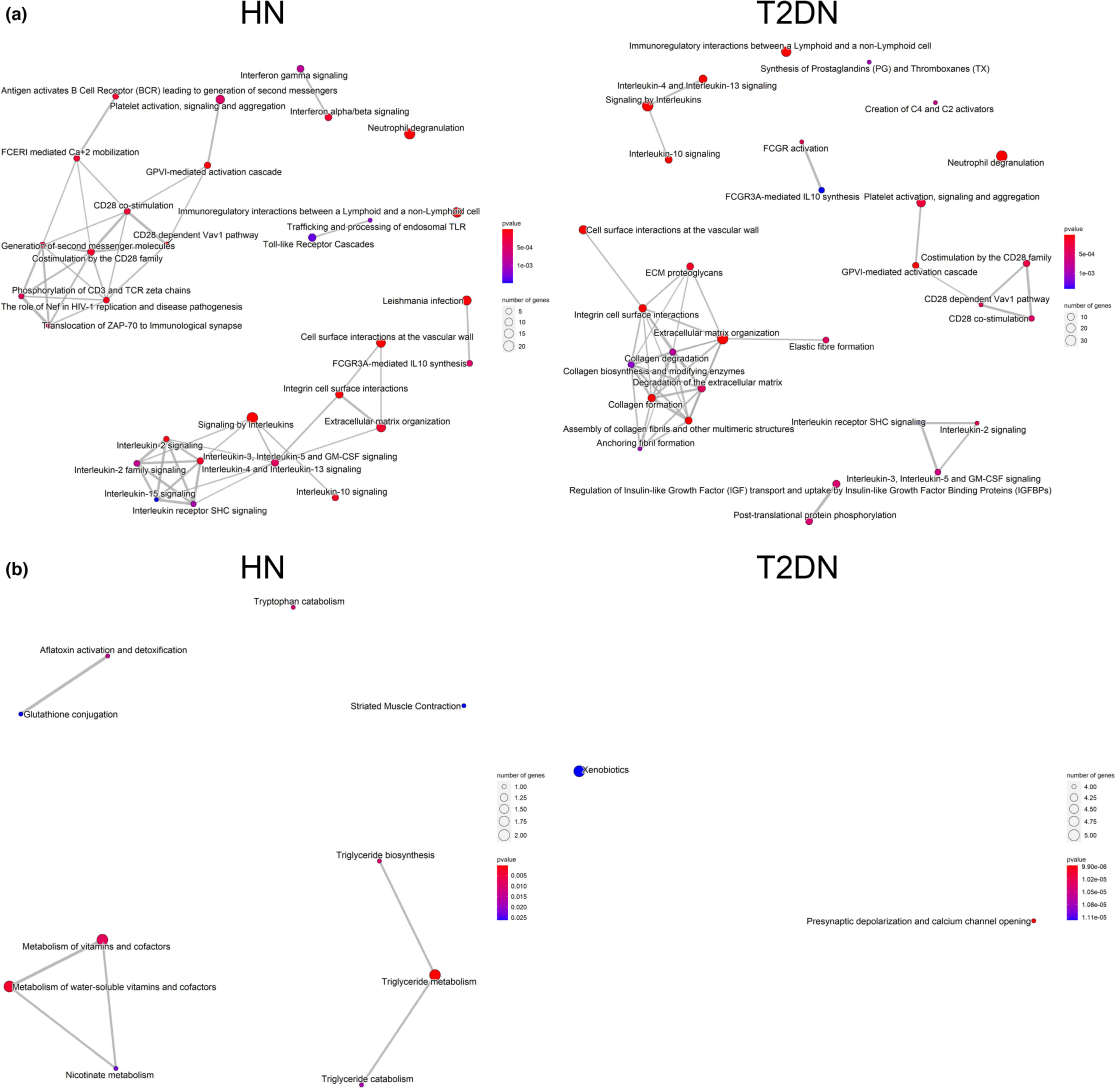
Pathway analysis of upregulated and downregulated genes in HN and T2DN. (a) Upregulated and (b) downregulated pathways in HN and T2DN determined by network analysis. The size of the nodes corresponds to the number of significantly expressed genes in each pathway, while the p‐values are represented by color (red is more significant, blue is less). Only significantly expressed genes (adj. *p* value < 0.05, log_2_ fold change >1) were used as input for the analysis. The pathway analysis was performed with ClusterProfiler v.4.1.

**FIGURE 3:**
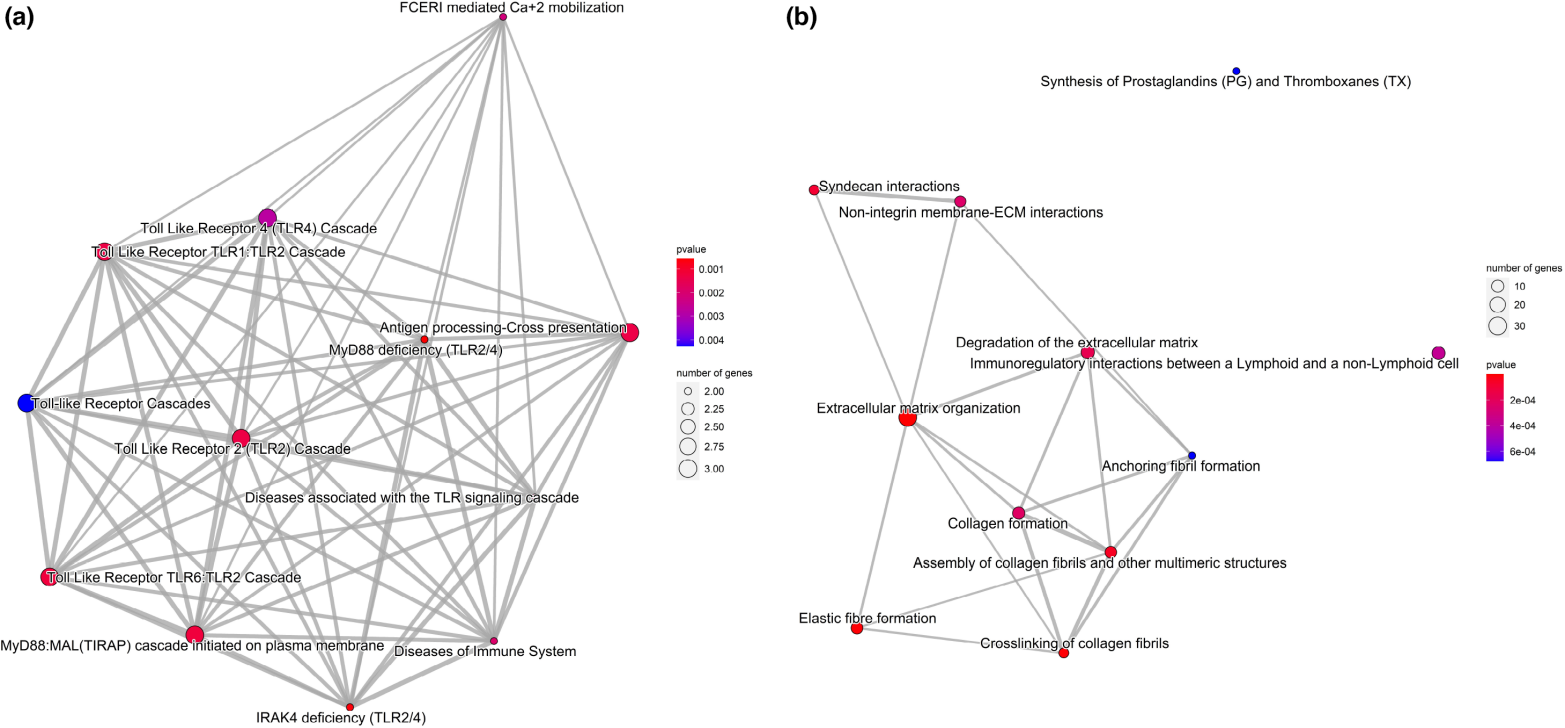
Network analysis of genes expressed only in HN or T2DN. (a) Network analysis generated based on significantly expressed genes in HN only and (b) upregulated significantly expressed genes in T2DN only. The size of the nodes corresponds to the number of significantly expressed genes in each pathway, while the *p*‐values are represented by color (red is more significant, blue is less). Only significantly expressed genes (adj. *p* value < 0.05, log_2_ fold change >1) were used as input for the analysis. The pathway analysis was performed with ClusterProfiler v4.1.

Pathway network analysis was performed on genes up‐ or downregulated in HN and T2DN specimens using ClusterProfiler (Yu et al., 2012) (Figure 2). The expression of similar gene networks was upregulated in both HN and T2DN tissues. However, overexpression of gene networks related to interleukin's signaling, lymphocyte activation, and CD28 co‐stimulation was predominantly, albeit not exclusively, detectable in HN. Genes involved in the extracellular matrix organization network were more evident in T2DN tissues (Figure 2a). Networks of genes downregulated in HN samples were mainly related to the metabolism of triglycerides and water‐soluble vitamins/cofactors, while in T2DN tissue, the only two downregulated pathways were xenobiotics and presynaptic depolarization and calcium channel opening (Figure 2b). Pathway analysis performed on genes differentially expressed to significant extents in either HN or T2DN revealed a distinct network of genes associated with toll‐like receptor cascade in HN and a network of genes related to ECM, collagen, and fibril formation in T2DN tissues (Figure 3).

### 3.4 Increased levels of T‐cells and related genes

**FIGURE 4:**
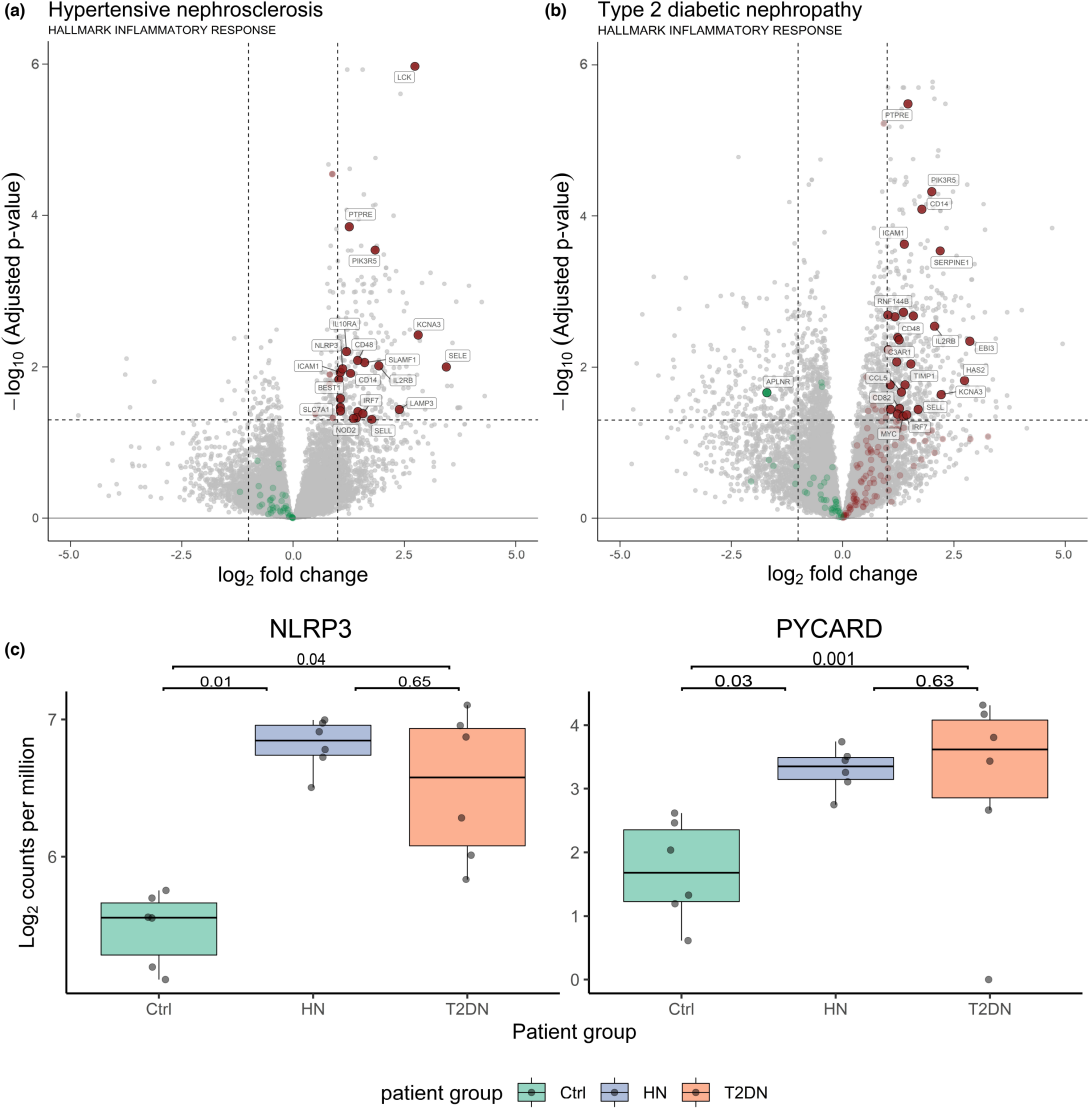
Upregulated proinflammatory gene‐ signature in HN and T2DN. (a) Volcano plots depicting upregulated and downregulated genes labeled as implicated with the inflammatory response in HN and (b) T2DN. (c) Boxplots of dysregulated genes of the inflammasome (*NLRP3* and *PYCARD*). Gene expression values are given as log_2_ counts per million, and significance values given as adjusted *p*‐values.

Volcano plots with a focus on inflammation were generated based on the Molecular Signatures Database (MSigDB) “Hallmark inflammatory response” (M5932) from the “Gene Set Enrichment analysis” of UCSD (USA) (https://www.gsea‐msigdb.org) (Figure 4a). In agreement with pathway analysis data, we observed a significant upregulation of a large number of genes associated with inflammatory responses (log_2_ FC >1; adjusted *p* < 0.05) in both disease entities (Figure 4a,b). In particular, expression of genes encoding NLR family pyrin domain‐containing 3 (*NLRP3*) and the apoptosis‐associated speck‐like protein containing a CARD (*PYCARD*), key initiators of inflammasome assembly, was significantly increased in both HN and T2DN, in comparison with normal tissues (Figure 4c).

**FIGURE 5:**
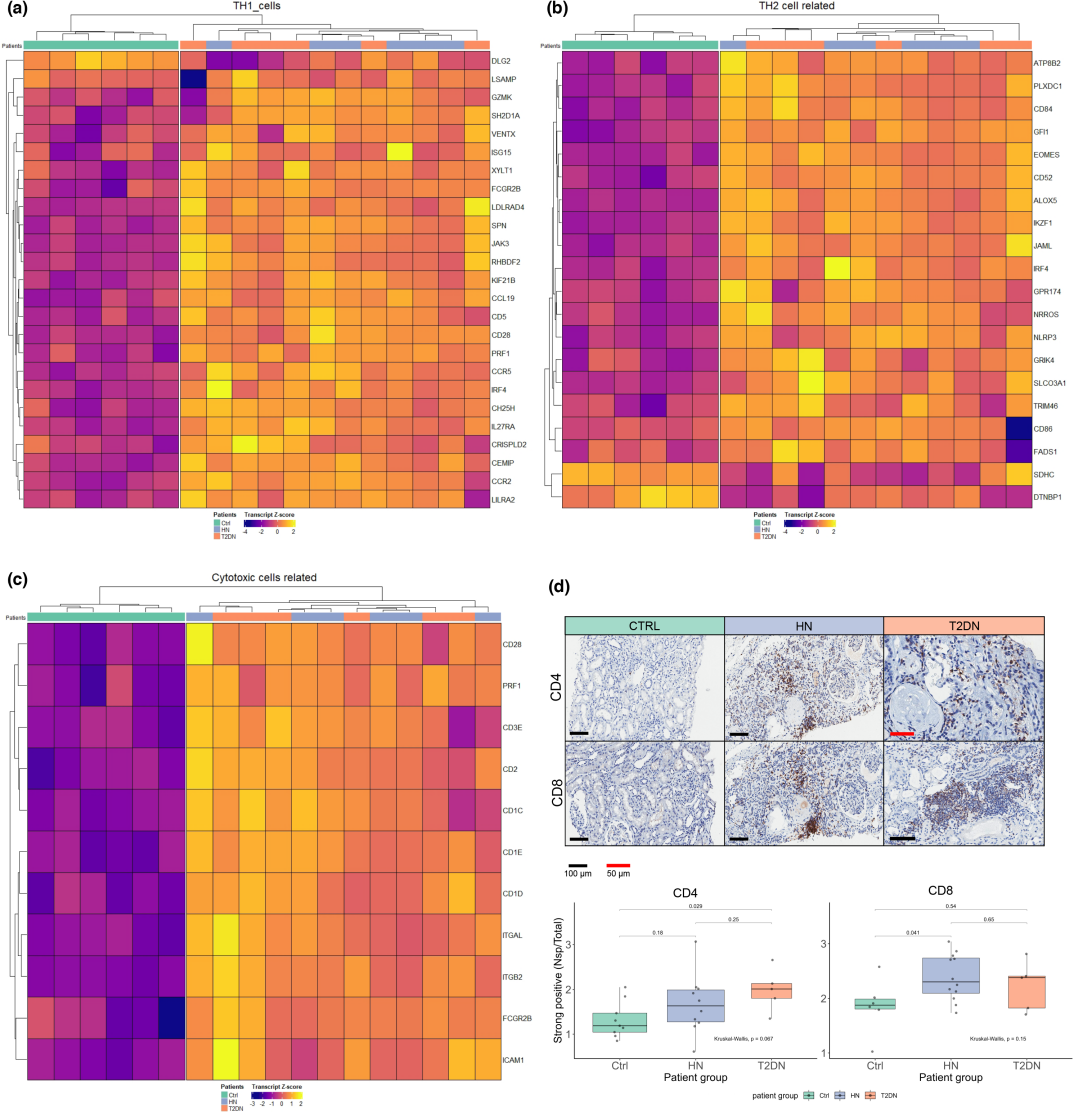
T‐cell and macrophage infiltration of HN and T2DN tissues. Hierarchical heatmap clustering shows increased expression of genes related to (a) T‐helper type 1 cells; (b) T‐helper type 2 cells; and (c) cytotoxic T‐cells. (d) Immunohistochemistry for CD4 and CD8 with boxplot showing Aperio ImageScope analysis of positive pixel count of strong positive pixels divided by total section area for each sample. The transcript *Z*‐score in the heatmaps is displayed as blue (lower score) and yellow (higher score). IHC scale bar 100 μm and 50 μm (shown in red), magnification 20×. *p*‐values from ImageScope analyses are based on the Kruskal–Wallis test.

The IPA tool retrieved the most enriched pathways in the gene expression data. The top five pathways identified by the IPA tool in HN and T2DN are listed in Tables 2 and 3, respectively. Notably, in both datasets, the highest expressed activation pathways were consistently associated with T‐helper (TH) cell activity, leukocyte extravasation signaling and phagosome formation. A full IPA report is included in Data S1. We performed hierarchical clustering of TH1‐ and TH2‐associated genes in both HN and T2DN diseased renal tissues (Figure 5a,b). Macrophages and cytotoxic (CD8^+^) cells are the main interaction partners of T helper cells. Accordingly, gene expression data analysis by hierarchical clustering showed a preferential overexpression of cytotoxic T cells‐associated gene signatures in HN and T2DN tissues compared to controls (Figure 5c). Gene expression profile findings could be validated, at the protein level, by the detection of CD4^+^ and CD8^+^ T‐cell infiltration in both HN and T2DN samples, notably surrounding sclerotic glomeruli (Figure 5d).

**TABLE 2:** Highest scoring canonical pathways in hypertensive nephrosclerosis tissues derived through ingenuity pathway analysis.

Pathway	*p*‐value	Overlap (%)
Th1 and Th2 activation pathway	2.68E‐13	11 (19/172)
Th2 pathway	7.29E‐13	12.4 (17/137)
Th1 pathway	1.92E‐11	12.3 (15/122)
Leukocyte extravasation signaling	1.85E‐10	8.8 (17/193)
Phagosome formation	2.89E‐10	4.5 (31/691)

*Note*: Name of the pathway, *p*‐value, and percentage of overlapping genes.

**TABLE 3:** Highest scoring canonical pathways in Type 2 diabetic nephropathy tissues derived through ingenuity pathway analysis.

Pathway	*p*‐value	Overlap (%)
Th1 and Th2 activation pathway	2.79E‐10	14.5 (25/172)
Th2 pathway	4.71E‐10	17.0 (23/135)
Phagosome formation	6.36E‐10	16.0 (24/150)
Th1 pathway	1.21E‐08	17.1 (21/123)
Leukocyte extravasation signaling	6.67E‐08	17.4 (19/109)

*Note*: Name of the pathway, *p*‐value, and percentage of overlapping genes.

### 3.5 Upregulated partial EMT signature

**FIGURE 6:**
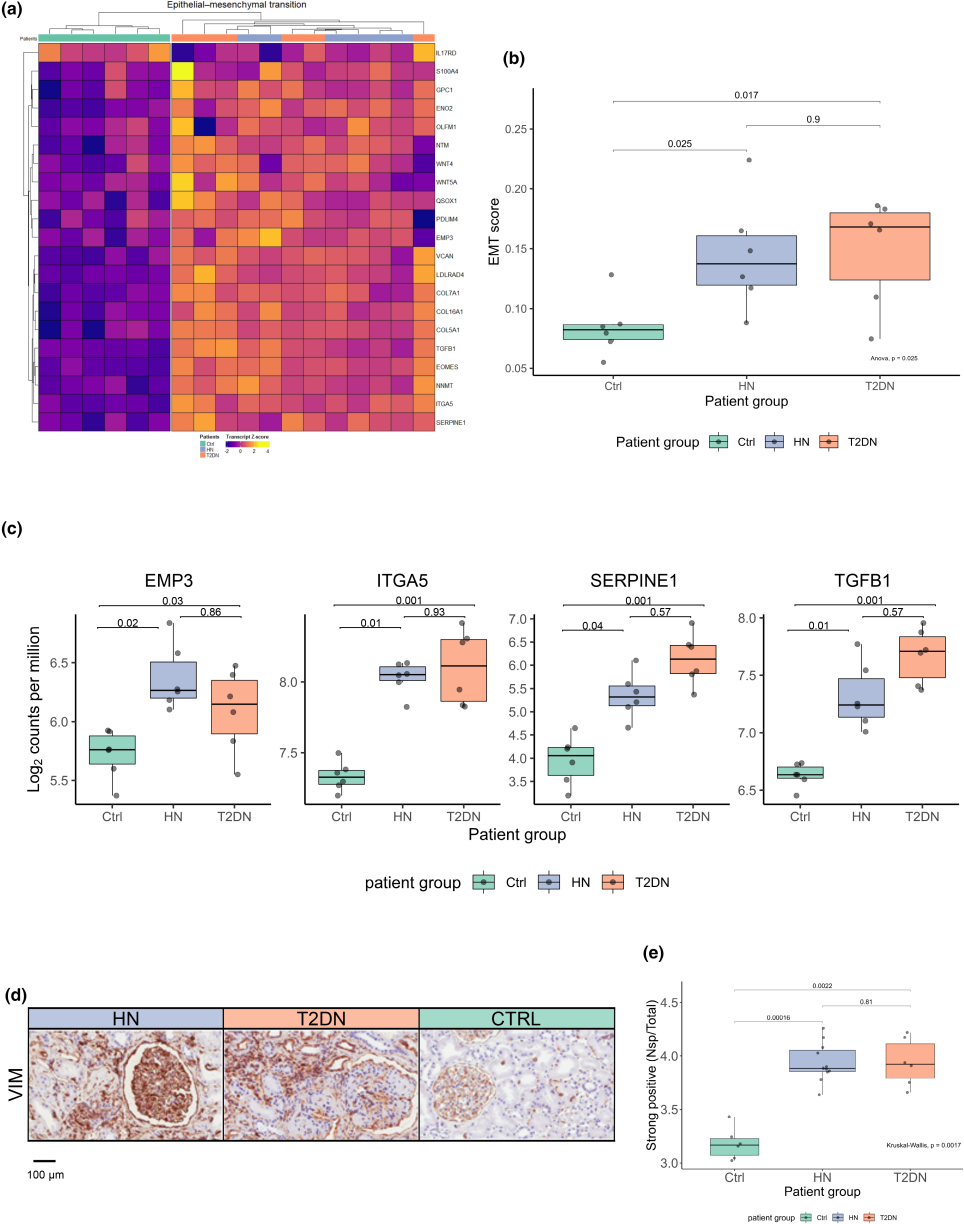
Epithelial‐to‐mesenchymal transition is a feature in hypertensive and T2DN nephropathy. (a) Hierarchical heatmap clustering using curated EMT genes as input. The transcript *Z*‐score in the heatmap was displayed as blue (lower score) and yellow (higher score). (b) Generic EMT score computed based on select EMT genes. EMT score in T2DN and HN = 0.17 and 0.14, respectively, control = 0.08. Significance *p*‐values are given as one‐way ANOVA. (c) The boxplots show selected genes related to p‐EMT (*EMP3*, *ITGA5*, *SERPINE1*, *TGBF1*) expressed in HN and T2DN. (d) IHC of vimentin with (e) boxlot showing Aperio ImageScope analysis of positive pixel count of strong positive pixels divided by total section area for each sample. Patient groups coloring: HN; violet, T2DN; orange, Ctrl; turquoise. patients: *n* = 6 per group. The expression values for the depicted genes in the boxplots were given as log_2_ counts per million. EMT genes for heatmap were obtained through the molecular signatures database, GS ID: M5930. The image analysis was performed with Aperio ImageScope. P‐values for ImageScope analysis are given as the Kruskal–Wallis test.

Chronic tissue inflammation is frequently associated with fibrosis in different tissues, including the kidney (Grande et al., 2015; Landolt et al., 2022; Lovisa et al., 2015). EMT then characterizes progression towards CKD differently (Carew et al., 2012; Stone et al., 2016). Therefore, we explored the differential expression of EMT‐related genes, as defined based on the “Hallmark EMT” gene set (systematic name: M5930) from the Molecular Signatures Database (MSigDB; (https://www.gsea‐msigdb.org/gsea/msigdb/index.jsp). Indeed, the expression of several EMT‐related genes was upregulated in both HN and T2DN (Figure 6a). To quantify these findings more reliably, we analyzed our data according to a previously described EMT score (Tan et al., 2014). The EMT score was based on the enrichment of 403 EMT‐related genes (Table S3). Although this method was originally developed for the analysis of malignant tissues, we observed that a significant increase was detectable in both HN and T2DN (~0.14 and ~ 0.17) as compared with control samples (~0.08, Figure 6b). Fourteen genes have previously been reported to be associated with p‐EMT (Puram et al., 2017). We found that the expression of four of these genes was upregulated in both HN and T2DN samples (Figure 6c). The expression of seven p‐EMT genes was upregulated, and one was downregulated in T2DN only (Figure S1). The expression of defined EMT markers was further investigated at the protein level by IHC. In particular, VIM, a Type 3 intermediate filament, is a critical component of the cytoskeleton of mesenchymal cells (Coulombe & Wong, 2004; Ivaska et al., 2007), and its expression is required for EMT development (Wang et al., 2018). IHC demonstrated increased VIM protein abundance in HN and T2DN biopsies compared to controls (Figure 6d,e).

### 3.6 Upregulated fibrosis and extracellular matrix remodeling gene signature

**FIGURE 7:**
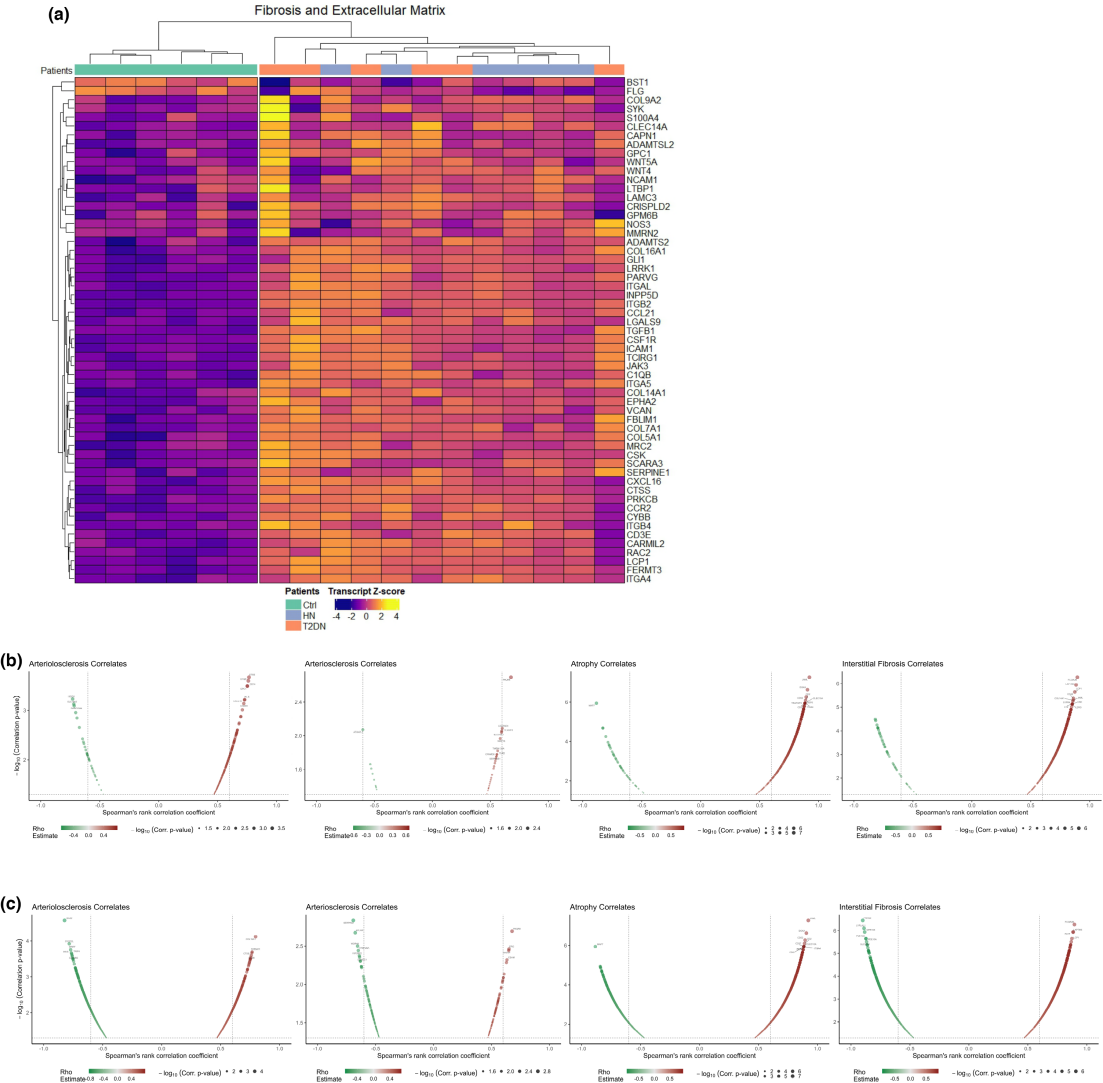
Extracellular matrix and tissue remodeling is elevated in the gene expression profiles of hypertensive and T2DN nephropathy. (a) Hierarchical heatmap clustering of genes related to the ECM, fibrosis, and collagen‐related genes. (b) Spearman's correlation plots showing HN genes and their correlations to morphological tissue changes including arteriolosclerosis, arteriosclerosis, tubular atrophy, and interstitial fibrosis. (c) Spearman's correlation plots showing T2DN genes and their correlations to morphological tissue changes including arteriolosclerosis, arteriosclerosis, tubular atrophy, and interstitial fibrosis. Patient groups coloring: HN; violet, T2DN; orange, Ctrl; turquoise. Gs Ids used as input for heatmaps: A: M25974, M18403, M15809, M26824, M17473, M26970, M3005, M631, M26976, M840, M587, M39652, M19176, M27376, M39652, M587, M26976, M14816; Genes involved in renal fibrosis from the FibroAtlas database v1.0 (https://biokb.ncpsb.org.cn/fibroatlas/index.php/Home/Help/), collagen genes from the HGNC (https://www.genenames.org), and from Gu et.al, 2020. For the correlation analysis, the *p* values are depicted as –log_10_.

CKD‐associated fibrosis is characterized by increased collagen production and ECM remodeling. Indeed, increased expression of a range of collagen, ECM‐related, ECM/tissue remodeling, and fibrosis genes was detectable in HN and T2DN tissues (Figure 7a). Genes used in Figure 7a were obtained from the FibroAtlas database (Liu et al., 2019), collagens annotated by the HGNC (https://www.genenames.org/) and genes involved in renal fibrosis from a recent comparative study on fibrosis (Gu et al., 2020). Further, we correlated gene expression values to degree of arteriolosclerosis, atherosclerosis, interstitial fibrosis, and tubular atrophy for significantly expressed HN (Figure 7b) and T2DN (Figure 7c). The complete list of correlates are available in Table S4.

### 3.7 AXL protein expression

The DEG data consistently showed the presence of both inflammation and partial EMT (p‐EMT) in both HN and T2DN tissue samples. Interestingly, in renal tissues, p‐EMT is both triggered and accompanied by inflammation. This could potentially create a harmful feedback loop if the primary inflammatory factors are not resolved (Kalluri & Weinberg, 2009).

AXL, a receptor tyrosine kinase on the cell surface and part of the TAM family plays a crucial role in regulating inflammation and EMT (Asiedu et al., 2014; Bellan et al., 2019; Lemke & Rothlin, 2008). It might be a promising therapeutic target for CKD (Landolt et al., 2022). When cleaved by a protease, the AXL protein generates a soluble extracellular domain that interacts with its ligand‐binding protein GAS6 (O'Bryan et al., 1995). Soluble AXL (sAXL) has recently been utilized as a serum biomarker for liver fibrosis and cirrhosis (Dengler et al., 2017), and has been associated with CKD (Lee et al., 2015). Furthermore, elevated sAXL levels have been observed in hemodialysis patients (Li et al., 2021).

**FIGURE 8:**
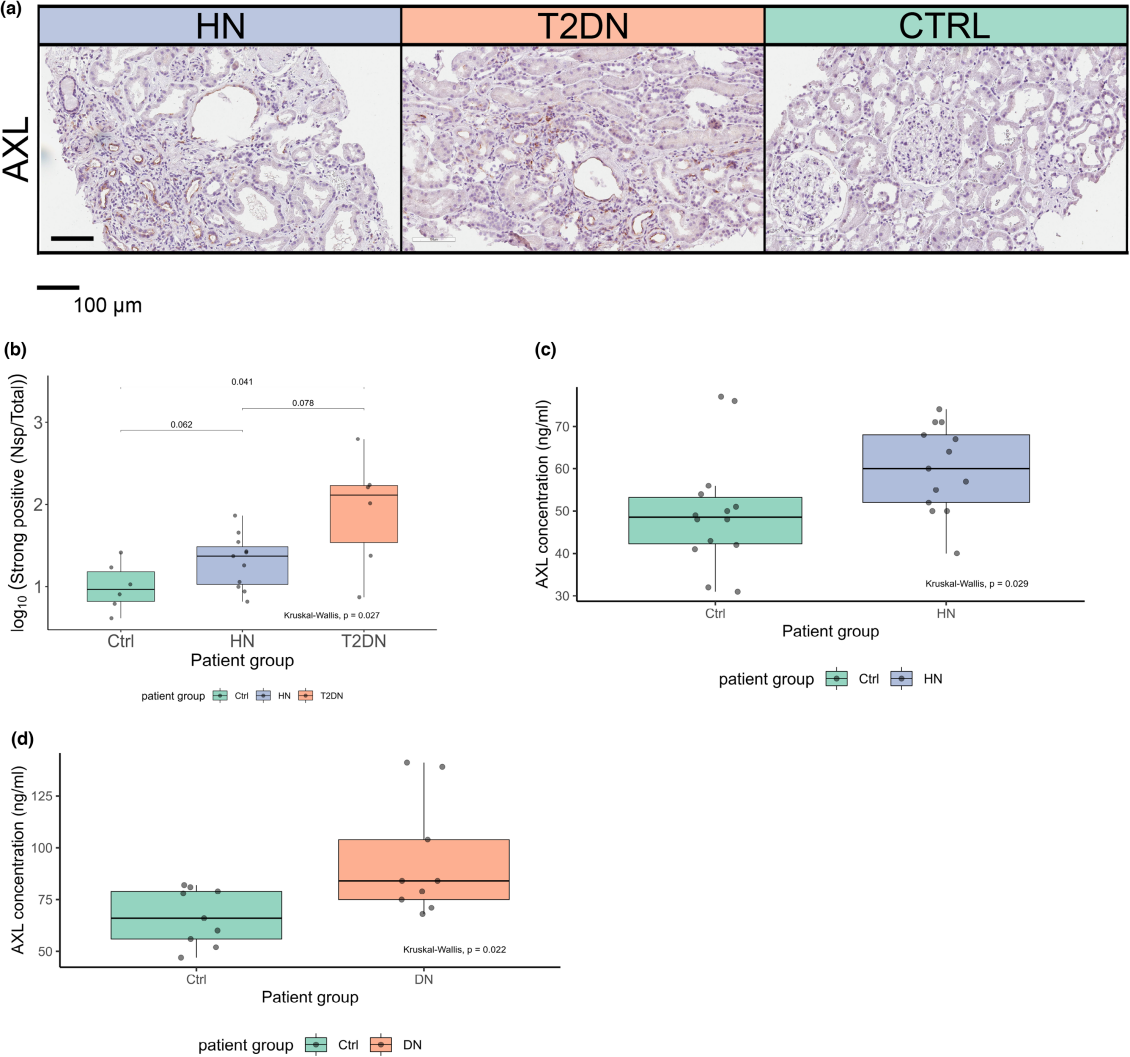
Elevated AXL Protein Levels in HN and T2DN Tissues and Serum. (a) AXL immunohistochemistry staining. (b) Ratio of strong positive pixels to total section area. (c) ELISA results for soluble AXL in serum from HN and control patients (*n* = 13 for HN, *n* = 14 for control). (d) ELISA results for serum from T2DN/T1DN and control patients (*n* = 7 for T2DN, *n* = 2 for T1DN, *n* = 9 for control). Measurements are presented in ng/mL. *p*‐values from ImageScope analyses are based on the Kruskal–Wallis test.

Importantly, cell surface AXL protein expression was identified through IHC in both HN and T2DN samples, but not in normal tissues (Figure 8a). The cohort included tissue samples from sequenced patients and four additional matched patients in the HN group. AXL's increased abundance was primarily seen in tubular regions of HN and T2DN, with only a few affected glomeruli in T2DN. AXL levels were significantly elevated only in T2DN tissues (Figure 8b). Data related to IHC protein expression are given in Table S5. In agreement with these findings, we observed significantly higher sAXL concentrations in the serum of adult HN patients (*n* = 14) compared to healthy controls (*n* = 14) (mean values = 59.9 ng/mL and 49.9 ng/mL, respectively; *p* = 0.038) (Figure 8c). Similar observations were also made upon testing of sera from T2DN patients (*n* = 9) versus controls (*n* = 9) (mean values = 37.6 ng/mL and 26.7 ng/mL, respectively; *p* = 0.024) (Figure 8d). Clinical data related to these cohorts are reported in Tables S6 and S7.

## 4 DISCUSSION

Our data indicate that these diseased tissues share a surprisingly high number of DEG compared to control kidney samples. Indeed, although T2DN specimens present more than twice the number of DEGs detectable in HN, pathways involved in either disease are primarily the same. More than 90 percent of those listed in the MsigDB database are shared by HN and T2DN tissues. Most importantly, the expression of signature genes associated with inflammation and p‐EMT appears to be similarly upregulated in both HN and T2DN specimens.

Inflammation is a driving factor in many diseases (Netea et al., 2017), and glomerular and tubulointerstitial inflammation is known to initiate and sustain the renal fibrotic process (Becker & Hewitson, 2000). Inflammation's critical role in T2DN has repeatedly been underlined (Hickey & Martin, 2018; Tang & Yiu, 2020). In murine and rat models of diabetic nephropathy, immune cell recruitment, and activation of resident immune cells result in increased production of proinflammatory cytokines, further favoring inflammation (Chen et al., 2019; Ito et al., 2021; Sassy‐Prigent et al., 2000). Moreover, a previous microarray study on microdissected glomeruli and tubules from patients with advanced DKD also provided evidence of increased expression of proinflammatory genes (Woroniecka et al., 2011).

The role of inflammation in HN has not been evaluated in close detail (Van Beusecum et al., 2022). However, data from experimental hypertension models also support a causal role of T cells in promoting increased blood pressure (Crowley et al., 2010; Guzik et al., 2007). CD4 T‐cell activity is increased in an angiotensin 2–induced hypertension model, and oxidative injury induces CD4 lymphocyte activation (Kirabo et al., 2014). Furthermore, inflammation may cause human hypertension also through the contribution of antigen‐presenting cells and a variety of other T‐cell subsets (Itani et al., 2016; Loperena et al., 2018; Norlander et al., 2018). Our data unravel a previously unsuspected similarity of gene expression profiles detectable in renal tissues from patients with HN and T2DN. As determined by IPA, the most upregulated pathways were associated with immune response and inflammation, including TH1 and TH2 activation, phagosome formation, and leukocyte extravasation signaling. Notably, although an antifibrotic effect of IFN‐γ has been reported in renal fibrosis experimental models (Oldroyd et al., 1999; Poosti et al., 2015), both TH1 and TH2 cells have previously been shown to facilitate the profibrotic process in the UUO model (Liu et al., 2012; Tapmeier et al., 2010). More recently, defective TH1 responses were reported to be associated with reduced collagen deposition in experimental models (Wen et al., 2019).

CKD has most frequently been shown to result from concurring inflammation and fibrosis (Webster et al., 2017). While myofibroblasts are the primary collagen depositors in renal fibrosis (Thiery et al., 2009), EMT is also involved in its progression and has received increasing attention in the last decade (Carew et al., 2012; Grande et al., 2015; Landolt et al., 2022; Lovisa et al., 2015). During classical EMT, stationary epithelial cells acquire mesenchymal characteristics (Kalluri & Weinberg, 2009; Thiery et al., 2009) with loss of cell–cell adhesion. However, in renal fibrosis, tubular epithelial cells (TECs) may gain a “mesenchymal” phenotype while remaining attached to the basement membrane. This process, referred to as “partial” EMT (p‐EMT) (Lovisa et al., 2016), leads to increased resistance to apoptosis and G2/M cell cycle arrest (Yang et al., 2010). In addition, p‐EMT stimulates tubulointerstitial myofibroblasts toward a profibrotic state (Huang & Susztak, 2016; Kalluri & Neilson, 2003; Wynn & Ramalingam, 2012). These processes interfere with the usual repair mechanisms in the damaged tissues and instead encourage repair methods that are not beneficial. Genes that correlate significantly with pathology scores often show a positive correlation with genes associated with inflammation. Examples of such genes include *JAML*, *DGKA*, and *CD52*. This correlation is expected, considering the extensive number of inflammation‐related genes that are upregulated. On the other hand, genes with the weakest correlation, such as *ENTPD5* and *HIBCH*, are involved in purine and valine metabolism, respectively.

Our data document the occurrence of p‐EMT in both HN and T2DN tissues, with concomitant modulation of the expression of several relevant genes (Gu et al., 2020; Liu et al., 2019). Consistent with the partial nature of the observed EMT, no decrease in E‐cadherin expression was observed in the tissues under investigation. Moreover, based on pathway analysis and comparison with previously published studies, p‐EMT appears to be less pronounced in HN than in T2DN tissues.

EMT of different extents is usually associated with augmented production of ECM proteins and tissue remodeling. In agreement with p‐EMT signature data, genes encoding collagens and ECM‐remodeling enzymes were upregulated in both HN and T2DN biopsies. While these results largely confirm data from a recent RNA‐Seq study on laser microdissected human kidney cortical interstitium in T2DN (Barwinska et al., 2021), they shed new light on ECM and remodeling in HN tissues.

The AXL receptor tyrosine kinase plays a pivotal role in cellular communication, transducing signals from the extracellular matrix (ECM) to the cytoplasm via adaptor proteins, notably GAS6 (Miller et al., 2016). One of the distinctive features of AXL is its propensity to induce an epithelial‐to‐mesenchymal transition (EMT) program (Boros et al., 2018; Gjerdrum et al., 2010). Moreover, AXL is deeply involved in modulating immune responses and inflammation across various tissues (Barcena et al., 2015; Bellan et al., 2019; Espindola et al., 2018; Lee et al., 2015; Lemke & Rothlin, 2008). A microarray study on T2DN revealed an upregulation of *AXL* in T2DN tubules and its downregulation in glomeruli (Woroniecka et al., 2011). Notably, our previous work demonstrated that bemcentinib, a selective AXL inhibitor, mitigates experimental renal fibrosis by reducing p‐EMT induction, inflammation, and downregulating genes associated with fibrosis in a unilateral ureteral obstruction (UUO) mouse model (Landolt et al., 2019).

We found an augmented AXL protein expression in both HN and T2DN tissues. This observation was further substantiated by the elevated sAXL levels in the sera of patients with HN and diabetes. Interestingly, despite the pronounced abundance of the AXL protein in both tissues and sera across diseases, *AXL* mRNA was predominantly overexpressed in T2DN renal tissues.

Drawing from other research, the Gas6/AXL pathway has been implicated in the pathogenesis of kidney diseases. A study documented the efficacy of CH5451098, a novel AXL inhibitor, in a mouse model of glomerular nephritis, revealing its potential in ameliorating kidney dysfunction by targeting EMT in tubular cells (Kurata et al., 2020). Similarly, another study showcased the potential of soluble AXL (sAXL) as a marker for assessing renal activity, histological response, and renal damage progression in lupus nephritis (Parodis et al., 2019).

We acknowledge the inherent limitations of our study. One of the major challenges lies in the nature of our bulk sequencing approach on renal tissues from HN and T2DN. Although it provides valuable insights, its resolution is not optimal for pinpointing specific cellular contributors to the observed gene expression changes. The term “EMT” implies a role of tubular cells in pro‐fibrotic pathways; however, our method might not accurately distinguish between various cells involved, such as activated fibroblasts or infiltrating immune cells. Techniques like laser capture microdissection (LCM) or spatial transcriptomics could offer a more precise cellular resolution. However, LCM demands a significant amount of tissue, and spatial transcriptomics remains a costly option. Additionally, our study is constrained by a relatively small patient cohort and the younger mean age of the control group compared to the diseased cohort. Even though we adjusted for patient age in our gene expression analysis, the patterns identified could still be influenced by age‐related factors (Sato & Yanagita, 2019). Another consideration is that our control biopsies, performed per clinical indications, might contain early structural damage not evident under standard light microscopy. Lastly, while our findings indicate a statistical significance for sAXL, this might not directly equate to clinical significance. Nonetheless, our perspective on sAXL is grounded in previous research highlighting its potential as a biomarker for renal diseases.

Our results support a close similarity of HN and T2DN transcriptomic signatures and emphasize shared pathological features, including inflammation associated with T‐cell immunity and p‐EMT. It is tempting to speculate that similar therapeutic strategies could benefit patients with either disease.

## AUTHOR CONTRIBUTIONS

OPN wrote the manuscript and performed the analysis. TA and HPN edited the manuscript, with TA also providing samples for ELISA. JF conducted ELISA experiments. LL handled RNA extraction, tissue cohort assembly, and co‐concieved the study. SL conducted histological examinations. HPM and PM secured funding and strategic input. AS managed the Ingenuity Pathway Analysis. OE contributed to the patient selection and co‐concieved the study. All authors have read and revised the manuscript and have approved the final version.

## FUNDING INFORMATION

This work was funded through an open project grant from the Western Norwegian Health Region (Helse Vest) awarded to Hans‐Peter Marti (project no. F‐12559), in addition to funding from the Broegelmann Foundation (Piotr Mydel).

## CONFLICT OF INTEREST STATEMENT

No author has any conflict of interest to report regarding this study.

## ETHICS STATEMENT

The study was conducted in accordance with the Declaration of Helsinki and approved by our local ethics committee REK vest (project approval numbers: REK vest 2013/553 and 609670).

## Supporting information


Data S1:
