# Supplementary material for: Transcriptomic analysis reveals partial epithelial–mesenchymal transition and inflammation as common pathogenic mechanisms in hypertensive nephrosclerosis and Type 2 diabetic nephropathy
Source: Physiol Rep. 2023 Oct 9;11(19):e15825. doi: 10.14814/phy2.15825 (PMC10562137; doi:10.14814/phy2.15825)
Supplement: Supplementary file 1 — Data S1: [file PHY2-11-e15825-s001.zip › T2DNvsCtrlDEGFCandadjP_IPAreport.pdf]

INGENUITY
PATHWAY ANALYSIS

Analysis Name: T2DNvsCtrlDEGFCandadjP - 2021-11-19 01:17 PM
Analysis Creation Date: 2021-11-19
Build version: exported
Content version: 68752261 (Release Date: 2021-09-06)

Experiment Metadata

Name	Value

Analysis Settings

Reference set: Ingenuity Knowledge Base (Genes Only)
Relationship to include: Direct and Indirect
Includes Endogenous Chemicals
Optional Analyses: My Pathways My List

Filter Summary:
Consider only molecules and/or relationships where
(species = Human OR Rat OR Uncategorized OR Mouse) AND
(confidence = Experimentally Observed) AND
(tissues/cell lines = Vascular smooth muscle cells OR A2780 OR INS-1 OR THP-1 OR Pituitary Gland OR COLO205 OR H460 OR HCT-15 OR Kidney cell lines not otherwise specified OR Megakaryocytes OR Epidermis OR Osteosarcoma Cell Lines not otherwise specified OR M14 OR NCI-H226 OR Choroid Plexus OR Retina OR Myeloma Cell Lines not otherwise specified OR Stem cells not otherwise specified OR U2OS OR

(c) 2000-2021 QIAGEN. All rights reserved.

Osteoblasts OR Breast Cancer Cell Lines not otherwise specified OR U937 OR Myeloid dendritic cells OR Immune cell lines not otherwise specified OR Uterus OR Mononuclear leukocytes not otherwise specified OR Plasmacytoid dendritic cells OR P19 OR HL-60 OR CD56bright NK cells OR Effector memory RA+ cytotoxic T cells OR NK cells not otherwise specified OR Purkinje cells OR Pyramidal neurons OR Mature monocyte-derived dendritic cells OR DU-145 OR Effector T cells OR Langerhans cells OR Other Pancreatic Cancer Cell Lines OR PBMCs OR Memory B cells OR Other T lymphocytes OR Eosinophils OR Substantia Nigra OR Liver OR Leukemia Cell Lines not otherwise specified OR Other Peripheral blood leukocytes OR T47-D OR Dermis OR Parietal Lobe OR CCRF-CEM OR Cervical cancer cell line not otherwise specified OR Sciatic Nerve OR Smooth muscle cells not otherwise specified OR KM-12 OR PC-12 cells OR Activated CD56bright NK cells OR Intraepithelial T lymphocytes OR Mesenchymal stem cells OR Other Kidney Cancer Cell Lines OR U87MG OR Microglia OR LNCaP cells OR Other Immune cell lines OR Hep3B OR Hepatoma Cell Lines not otherwise specified OR Other Pheochromocytoma cell lines OR Astrocytes OR Salivary Gland OR Other CNS Cell Lines OR MDA-MB-361 OR HeLa OR Tissues and Primary Cells not otherwise specified OR Jurkat OR Th1 cells OR Macrophage Cancer Cell Lines not otherwise specified OR HOP-62 OR SK-OV-3 OR Effector memory cytotoxic T cells OR Activated helper T cells OR Cerebral Cortex OR J774 OR Other Bone marrow cells OR Melanoma Cell Lines not otherwise specified OR BDCA-3+ dendritic cells OR NIH/3T3 cells OR Peripheral blood lymphocytes OR Cerebral Ventricles OR Neurons not otherwise specified OR Dorsal Root Ganglion OR Monocytes not otherwise specified OR Gray Matter OR Other Breast Cancer Cell Lines OR Heart OR SF-295 OR Cortical neurons OR Pro-B lymphocytes OR ACHN OR WEHI-231 OR Testis OR A498 OR Peritoneal macrophages OR Kidney Cancer Cell Lines not otherwise specified OR Teratocarcinoma Cell Lines not otherwise specified OR Kidney OR HCC-2998 OR Cells not otherwise specified OR Peripheral blood leukocytes not otherwise specified OR Monocyte-derived macrophage OR Other Colon Cancer Cell Lines OR Other Granulocytes OR Forestomach OR B lymphocytes not otherwise specified OR MDA-N OR Epithelial cells not otherwise specified OR Other Neurons OR SF-539 OR Neutrophils OR OVCAR-5 OR Thalamus OR Activated Vd2 Gamma-delta T cells OR Cardiomyocytes OR Activated Vd1 Gamma-delta T cells OR Other Cervical cancer cell line OR Peripheral blood monocytes OR Adrenal Gland OR Th2 cells OR OVCAR-3 OR Other Nervous System OR Striatum OR SW-620 OR Other Leukemia Cell Lines OR Stomach OR IGROV1 OR Other Cell Line OR HMC-1 OR HuH7 OR Prostate Cancer Cell Lines not otherwise specified OR Other Lymphoma Cell Lines OR Other Macrophage Cancer Cell Lines OR Ovarian Cancer Cell Lines not otherwise specified OR Crypt OR Calvaria OR Dendritic cells not otherwise specified OR PANC-1 OR Cartilage Tissue OR CD4+ T-lymphocytes OR SN12C OR Brain OR Other Hepatoma Cell Lines OR UO-31 OR Lymphocytes not otherwise specified OR SW-480 OR Th17 cells OR SK-MEL-28 OR Pancreatic Cancer Cell Lines not otherwise specified OR Natural T-regulatory cells OR HCT-116 OR Other Myeloma Cell Lines OR Cerebellum OR Skin OR Other Lymphocytes OR Esophagus OR Other NK cells OR MALME-3M OR Oocytes OR Other Smooth muscle cells OR SR OR Trachea OR HEL OR Other Endothelial cells OR Microvascular endothelial cells OR J-774A.1 OR NCI-H522 OR Vd1 Gamma-delta T cells OR Swiss 3T3 cells OR A375 OR Olfactory Bulb OR Prostate Gland OR Other Lung Cancer Cell Lines OR Other Memory T lymphocytes OR RBL-2H3 OR Granulosa cells OR CAKI-1 OR Colon Cancer Cell Lines not otherwise specified OR NB4 OR Bone

(c) 2000-2021 QIAGEN. All rights reserved.

INGENUITY PATHWAY ANALYSIS

marrow cells not otherwise specified OR Lens OR Other Stem cells OR Bone marrow-derived macrophages OR Naive helper T cells OR MCF7 OR Caudate Nucleus OR T lymphocytes not otherwise specified OR LOX IMVI OR Embryonic stem cells OR Monocyte-derived dendritic cells not otherwise specified OR BT-549 OR Melanocytes OR HUVEC cells OR Other Mononuclear leukocytes OR UACC-62 OR Immune cells not otherwise specified OR Ventricular Zone OR Cytotoxic T cells OR Nucleus Accumbens OR Other Neuroblastoma Cell Lines OR Granule Cell Layer OR Medulla Oblongata OR MG-63 OR Granule cells OR RPMI-8266 OR Splenocytes OR HOP-92 OR U251 OR Granulocytes not otherwise specified OR CNS Cell Lines not otherwise specified OR Beta islet cells OR Nervous System not otherwise specified OR OVCAR-8 OR EKVX OR MOLT-4 OR Trigeminal Ganglion OR K-562 OR Other Teratocarcinoma Cell Lines OR OVCAR-4 OR Hepatocytes OR Organ Systems not otherwise specified OR Fibroblast cell lines not otherwise specified OR Other Melanoma Cell Lines OR Large Intestine OR 293 cells OR Other Fibroblast cell lines OR MEF cells OR CD34+ cells OR NT2/D1 OR White Matter OR Cornea OR Pancreas OR BA/F3 OR Spinal Cord OR Other Dendritic cells OR Skeletal Muscle OR MDA-MB-468 OR Other Immune cells OR Hematopoietic progenitor cells OR HT29 OR Immature monocyte-derived dendritic cells OR NCI-ADR-RES OR SF-268 OR Plasma cells OR SK-MEL-2 OR Chondrocytes OR Blood platelets OR MDA-MB-435 OR PC-3 OR Central memory cytotoxic T cells OR Other Monocytes OR Other Monocyte-derived dendritic cells OR Other Organ Systems OR Adipose OR Bone marrow-derived dendritic cells OR Fibroblasts OR A549-ATCC OR Brainstem OR HepG2 OR Placenta OR Corpus Callosum OR Other Cells OR Small Intestine OR Other Osteosarcoma Cell Lines OR RAW 264.7 OR Pheochromocytoma cell lines not otherwise specified OR Smooth Muscle OR Macrophages not otherwise specified OR Lung Cancer Cell Lines not otherwise specified OR SK-N-SH OR U266 OR Mast cells OR CD56dim NK cells OR HS 578T OR Amygdala OR BT-474 OR Lymphoma Cell Lines not otherwise specified OR RXF-393 OR Bladder OR Other Ovarian Cancer Cell Lines OR Subventricular Zone OR NCI-H332M OR Neuroblastoma Cell Lines not otherwise specified OR Stromal cells OR Naive B cells OR Murine NKT cells OR Thymus OR Endothelial cells not otherwise specified OR 3T3-L1 cells OR Adipocytes OR Mammary Gland OR Other Prostate Cancer Cell Lines OR Caco2 cells OR Hippocampus OR Activated CD56dim NK cells OR TK-10 OR Vd2 Gamma-delta T cells OR Other Macrophages OR SNB-75 OR Spleen OR BDCA-1+ dendritic cells OR Other Tissues and Primary Cells OR NCI-H23 OR Putamen OR MDA-MB-231 OR 786-0 OR Thyroid Gland OR Lung OR Lymph node OR Ovary OR Pre-B lymphocytes OR SK-MEL-5 OR Keratinocytes OR Other Kidney cell lines OR Other B lymphocytes OR Cos-7 cells OR Hypothalamus OR Thymocytes OR Central memory helper T cells OR Other Epithelial cells OR RKO OR Min6 OR UACC-257 OR Sertoli cells OR Effector memory helper T cells OR Memory T lymphocytes not otherwise specified OR Cell Line not otherwise specified) AND
(mol. types = biologic drug OR canonical pathway OR chemical - endogenous mammalian OR chemical - endogenous non-mammalian OR chemical - kinase inhibitor OR chemical - other OR chemical - protease inhibitor OR chemical drug OR chemical reagent OR chemical toxicant OR complex OR cytokine OR disease OR enzyme OR function OR fusion gene/product OR G-protein coupled receptor OR group OR growth factor OR ion channel OR kinase OR ligand-dependent nuclear receptor OR mature microRNA OR microRNA OR other OR peptidase OR phosphatase OR transcription regulator OR translation regulator OR transmembrane receptor OR transporter) AND

(c) 2000-2021 QIAGEN. All rights reserved.

(data sources = An Open Access Database of Genome-wide Association Results OR BIND OR BioGRID OR Catalogue Of Somatic Mutations In Cancer (COSMIC) OR Chemical Carcinogenesis Research Information System (CCRIS) OR Clinical Genome Resource (ClinGen) OR ClinicalTrials.gov OR ClinVar OR Cognia OR DIP OR DrugBank OR Gene Ontology (GO) OR GVK Biosciences OR Hazardous Substances Data Bank (HSDB) OR HumanCyc OR Ingenuity Expert Findings OR Ingenuity ExpertAssist Findings OR IntAct OR Interactome studies OR MIPS OR miRBase OR miRecords OR Mouse Genome Database (MGD) OR Obesity Gene Map Database OR Online Mendelian Inheritance in Man (OMIM) OR TarBase OR TargetScan Human)

Top Canonical Pathways

Name	p-value	Overlap
**Th1 and Th2 Activation Pathway**	2.79E-10	14.5 % 25/172
**Th2 Pathway**	4.71E-10	16.1 % 22/137
**Phagosome Formation**	6.36E-10	8.0 % 55/691
**Th1 Pathway**	1.21E-08	15.6 % 19/122
**Leukocyte Extravasation Signaling**	6.67E-08	11.9 % 23/193

Top Upstream Regulators

Upstream Regulators

Name	p-value	Predicted Activation
**lipopolysaccharide**	4.48E-24	Activated
**IFNG**	2.00E-20	Activated
**TNF**	1.03E-18	Activated
**AHR**	2.08E-16	

(c) 2000-2021 QIAGEN. All rights reserved.

INGENUITY PATHWAY ANALYSIS

**semaxinib**	1.39E-15	Activated

Causal Network

Name	p-value	Predicted Activation
**monophosphoryl lipid A**	1.59E-27	Activated
**APOC1**	4.87E-27	Activated
**LY96**	7.43E-26	Activated
**REL/RELA/RELB**	8.92E-26	
**AGTRAP**	1.24E-25	Inhibited

Top Diseases and Bio Functions

Diseases and Disorders

Name	p-value range	# Molecules
**Cancer**	8.64E-11 - 3.81E-60	748
**Organismal Injury and Abnormalities**	1.02E-10 - 3.81E-60	751
**Dermatological Diseases and Conditions**	3.18E-12 - 4.87E-51	610
**Endocrine System Disorders**	5.96E-11 - 3.93E-40	673
**Gastrointestinal Disease**	5.96E-11 - 1.33E-38	699

Molecular and Cellular Functions

(c) 2000-2021 QIAGEN. All rights reserved.

INGENUITY PATHWAY ANALYSIS

Name	p-value range	# Molecules
Cellular Development	7.98E-11 - 8.10E-44	198
Cellular Growth and Proliferation	7.98E-11 - 8.10E-44	212
Cell-To-Cell Signaling and Interaction	1.06E-10 - 8.37E-39	237
Cellular Function and Maintenance	1.06E-10 - 5.32E-37	234
Cellular Movement	3.77E-11 - 7.36E-33	247

Physiological System Development and Function

Name	p-value range	# Molecules
Hematological System Development and Function	1.06E-10 - 8.10E-44	279
Lymphoid Tissue Structure and Development	7.98E-11 - 8.10E-44	227
Hematopoiesis	6.57E-11 - 6.94E-41	155
Tissue Development	8.99E-12 - 6.94E-41	195
Tissue Morphology	9.85E-11 - 5.18E-40	251

Top Tox Functions

Assays: Clinical Chemistry and Hematology

Name	p-value range	# Molecules
Increased Levels of LDH	5.81E-03 - 8.53E-05	7
Decreased Levels of Albumin	1.78E-01 - 1.28E-04	3

(c) 2000-2021 QIAGEN. All rights reserved.

**Increased Levels of AST**	1.23E-01 - 6.10E-04	4
**Increased Levels of ALT**	2.29E-01 - 7.79E-03	5
**Increased Levels of Red Blood Cells**	2.40E-02 - 2.40E-02	8

Cardiotoxicity

Name	p-value range	# Molecules
**Cardiac Infarction**	1.00E00 - 1.25E-08	34
**Cardiac Arteriopathy**	2.04E-01 - 2.07E-06	34
**Cardiac Arrythmia**	1.00E00 - 1.73E-05	29
**Cardiac Enlargement**	1.00E00 - 1.88E-04	44
**Cardiac Inflammation**	5.85E-01 - 3.12E-04	14

Hepatotoxicity

Name	p-value range	# Molecules
**Liver Hyperplasia/Hyperproliferation**	1.00E00 - 2.11E-15	370
**Liver Inflammation/Hepatitis**	4.79E-01 - 1.38E-10	48
**Hepatocellular carcinoma**	1.00E00 - 3.56E-08	114
**Liver Damage**	3.45E-01 - 6.83E-08	28
**Liver Cirrhosis**	1.78E-01 - 2.20E-05	28

Nephrotoxicity

(c) 2000-2021 QIAGEN. All rights reserved.

INGENUITY PATHWAY ANALYSIS

Name	p-value range	# Molecules
**Renal Inflammation**	2.26E-01 - 1.08E-09	45
**Renal Nephritis**	2.26E-01 - 1.08E-09	45
**Kidney Failure**	2.78E-01 - 1.96E-09	35
**Renal Damage**	3.45E-01 - 1.36E-06	33
**Glomerular Injury**	5.57E-01 - 2.97E-06	43

Top Regulator Effect Networks

ID	Regulators	Disease & Functions	Consistency Score
**1**	Nr1h	Cell movement	3.606
**2**	Nr1h	Leukocyte migration	3.606
**3**	Nr1h	Migration of cells	3.606
**4**	BHLHE40	Phagocytosis of cells	3.464
**5**	Nr1h	Cell movement of leukocytes	3.317

Top Networks

ID	Associated Network Functions	Score
**1**	Cancer, Cell Death and Survival, Cellular Development	42
**2**	Cancer, Organismal Injury and Abnormalities, Dermatological Diseases and Conditions	40

(c) 2000-2021 QIAGEN. All rights reserved.

**3**	Cancer, Organismal Injury and Abnormalities, Reproductive System Disease	36
**4**	Antigen Presentation, Lipid Metabolism, Small Molecule Biochemistry	33
**5**	Infectious Diseases, Cell Morphology, Hematological Disease	33

Top Tox Lists

Name	p-value	Overlap
**Hepatic Fibrosis**	1.36E-09	10.1 % 36/356
**Hepatic Stellate Cell Activation**	9.57E-08	28.6 % 10/35
**Genes associated with Chronic Allograft Nephropathy (Human)**	1.51E-07	38.1 % 8/21
**Increases Liver Damage**	2.55E-06	14.4 % 14/97
**Increases Renal Damage**	5.91E-06	13.5 % 14/104

Top My Lists

Top My Pathways

Top Analysis-Ready Molecules

(c) 2000-2021 QIAGEN. All rights reserved.

Expr Fold Change

Molecules	Expr. Value	Chart
**PLEKHN1**	↑ 49.308	
**SLC14A2**	↑ 30.721	
**CH25H**	↑ 26.088	
**WNT4**	↑ 16.261	
**CD79A**	↑ 13.433	
**CD1E**	↑ 12.963	
**SLPI**	↑ 12.936	
**ADGRG2**	↑ 10.985	
**TRIM29**	↑ 10.871	
**CLEC4E**	↑ 10.846	

Expr Fold Change

Molecules	Expr. Value	Chart
**LHCGR**	↓ -118.269	
**TNNI1**	↓ -36.204	
**PLIN1**	↓ -34.907	
**SLC7A10**	↓ -26.016	
**SRRM4**	↓ -24.639	
**USP6**	↓ -22.577	
**LPA**	↓ -18.906	
**RNF212B**	↓ -17.585	
**PCOLCE2**	↓ -14.461	

(c) 2000-2021 QIAGEN. All rights reserved.

INGENUITY PATHWAY ANALYSIS

**ALB**	↓	-14.099

(c) 2000-2021 QIAGEN. All rights reserved.

INGENUITY PATHWAY ANALYSIS
