# Supplementary figures and images for: Transcriptomic analysis reveals partial epithelial–mesenchymal transition and inflammation as common pathogenic mechanisms in hypertensive nephrosclerosis and Type 2 diabetic nephropathy
Source: Physiol Rep. 2023 Oct 9;11(19):e15825. doi: 10.14814/phy2.15825 (PMC10562137; doi:10.14814/phy2.15825)

## Partial EMT genes differentially expressed in T2DN

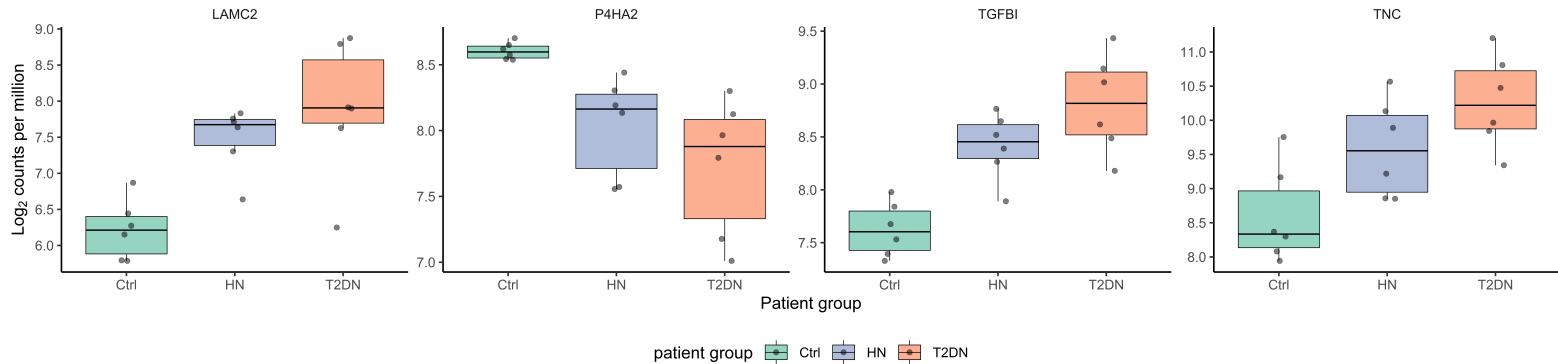

Supplement: Supplementary file 1 — Data S1: [file PHY2-11-e15825-s001.zip › figure_S1.pdf]
